# Implementation Lessons and Policy Implications of Same-Day Antiretroviral Therapy Initiation: Insights from the ART Same-Day Counselling and Initiation (ASCI) SOP in South Africa

**DOI:** 10.3390/ijerph23030378

**Published:** 2026-03-17

**Authors:** Siyakudumisa Nontamo, Gabriel Tchuente Kamsu, Nomboniso Agrinette Madolo, Eugene Jamot Ndebia

**Affiliations:** 1 Department of Nursing, Faculty of Medicine and Health Sciences, Walter Sisulu University, Mthatha 5100, South Africa; amadolo@wsu.ac.za; 2 Department of Human Biology, Faculty of Medicine and Health Sciences, Walter Sisulu University, Mthatha 5100, South Africa; gkamsu-tchuente@wsu.ac.za (G.T.K.); endebia@wsu.ac.za (E.J.N.)

**Keywords:** same-day ART initiation, antiretroviral therapy, pyschosocial suppport, patient navigation, retention in HIV care, viral suppression

## Abstract

**Highlights:**

**Public health relevance—How does this work relate to a public health issue?**

- Same-day ART initiation is recommended globally as a strategy to improve linkage to treatment in high-burden settings such as South Africa.
- Retention in care and viral HIV viral load suppression remain suboptimal, highlighting the need for a fortified patient-centered initiation model in South Africa.

**Public health significance—Why is this work of significance to public health?**

- The ASCI SOP improved retention in care (83% vs. 72%) and viral suppression (81% vs. 69%) compared with the standard FTIC model.
- Integrating patient navigation, psychosocial support, and community follow-up addresses readiness, stigma, disclosure, and system-level barriers affecting treatment outcomes.

**Public health implications—What are the key implications or messages for practitioners, policy makers and/or researchers in public health?**

- Scaling up structured same-day initiation models that include navigation, psychosocial and community-based support can strengthen HIV treatment outcomes nationally.
- Policymakers should integrate standardized readiness assessments and multidisciplinary support teams into routine HIV service delivery to minimize early disengagement of treatment.

**Abstract:**

Same-day antiretroviral therapy (ART) initiation (SDI) is globally recommended to improve ART uptake. However, retention in care and viral suppression in South Africa remain suboptimal. This study evaluated the experiences of healthcare providers, patients, and community stakeholders in implementing the ART Same-day Counselling and Initiation (ASCI) Standard Operating Procedure (SOP), focusing on facilitators, barriers, and policy implications for improving HIV treatment outcomes. Using implementation frameworks, qualitative data from providers, patients, and community structures were analyzed alongside findings from a randomized controlled trial involving 142 newly diagnosed individuals initiated on ART on the same day as diagnosis. Evaluation of the ASCI SOP demonstrated improved six-month outcomes compared with standard initiation: retention in care (83% vs. 72%), viral suppression (81% vs. 69%, *p* = 0.04), and reduced loss to follow-up (17% vs. 28%, *p* = 0.05), with no significant mortality difference. These gains were linked to structured psychosocial support, patient navigation, and community follow-up. Key facilitators included multidisciplinary collaboration, psychosocial support, and community engagement, while major barriers involved healthcare system overload, patient-level challenges, and lack of standardized tools to assess treatment readiness. Policy reform to scale up the ASCI SOP should emphasize interdisciplinary support, consistent monitoring, and integration within national health systems. Overall, the preliminary evidence suggests that implementing the ASCI SOP model improved same-day ART uptake, retention, and viral suppression. Expanding this model to other provinces could strengthen HIV program performance and accelerate progress toward South Africa’s treatment goals.

## 1. Introduction

The global HIV response has made significant progress towards the Joint United Nations Programme on HIV/AIDS (UNAIDS) 95-95-95 targets, which aim to ensure that 95% of people living with HIV (PLHIV) know their status, 95% of those diagnosed initiate antiretroviral therapy (ART), and 95% of those on treatment achieve viral suppression by 2030 [1]. Same-day initiation (SDI) of ART has emerged as a key strategy to accelerate progress toward these goals. By reducing delays between diagnosis and treatment initiation, SDI aims to minimize pre-treatment attrition, improve linkage to care, and enhance early viral suppression outcomes [2,3].

Evidence from randomized trials and programmatic studies across sub-Saharan Africa demonstrates that SDI can increase ART uptake; however, its impact on long-term retention and adherence has been mixed [4,5,6]. While some studies report improved linkage and reduced early attrition, others highlight challenges such as poor patient readiness, psychosocial distress, stigma, and subsequent loss to follow-up [7,8]. These findings underscore that while SDI addresses structural barriers to timely treatment, it does not automatically resolve the psychosocial and community-level challenges that shape adherence and continuity of care.

South Africa, home to the world’s largest HIV treatment program, adopted national guidelines in 2016 recommending SDI for clinically eligible patients [9]. The policy was designed to expedite the initiation of ART and minimize loss to follow-up between diagnosis and treatment initiation. However, implementation has faced challenges, particularly in high-burden and resource-limited settings such as the Eastern Cape Province. Health system constraints, patient-level barriers, and the absence of standardized readiness assessment tools have contributed to suboptimal retention and viral suppression outcomes under the current Fast-Track Initiation and Counselling (FTIC) model [10,11]. These challenges underscore the need for enhanced approaches that extend beyond the biomedical act of prescribing ART and instead integrate psychosocial, navigational, and community-based support mechanisms. The ART Same-day Counselling and Initiation (ASCI) Standard Operating Procedure (SOP) was developed as a structured enhancement to the FTIC model. The ASCI SOP combines same-day ART initiation with systematic psychosocial counselling, peer navigation, and community follow-up, with the aim of addressing readiness gaps, supporting adherence, and reducing early disengagement from care.

The rationale for ASCI is grounded in evidence that multi-layered interventions, incorporating psychosocial care, patient navigation, and community engagement, are more effective in sustaining long-term treatment outcomes than clinic-based counselling alone [12,13]. By embedding structured follow-up schedules and leveraging ward-based outreach teams, the ASCI SOP aims to establish a patient-centered pathway that addresses both clinical and social determinants of patient retention. The objective of this paper is to analyse the implementation lessons of the ASCI SOP in a high-burden district of South Africa, compare outcomes with the FTIC model, and draw policy implications for scaling up differentiated models of same-day ART initiation in resource-limited settings.

## 2. Methods

### 2.1. Study Design

This study adopted a mixed-methods design integrating a pragmatic, feasibility-driven randomized controlled trial (RCT) with qualitative implementation research. The RCT component was designed to evaluate the effectiveness of the ASCI SOP under real-world conditions in a resource-limited, high-burden district, rather than to detect small effect sizes under ideal trial conditions. Accordingly, the sample size was determined by clinic throughput, staffing capacity, and ethical considerations related to same-day ART initiation roll-out. The study was powered to detect moderate-to-large differences in outcomes; therefore, non-significant findings should be interpreted cautiously. A randomized controlled trial (RCT) was conducted to compare treatment outcomes among newly diagnosed HIV-positive individuals initiated on antiretroviral therapy (ART) under the ASCI SOP versus the existing Fast-Track Initiation and Counselling (FTIC) model. The RCT was complemented by implementation science frameworks, qualitative interviews, and community-level data collection to contextualize facilitators and barriers influencing implementation effectiveness.

### 2.2. Study Setting and Population

The study employed a random sampling of eligible participants from newly diagnosed HIV-positive individuals attending selected primary healthcare facilities in the O.R. Tambo District, Eastern Cape. The RCT population consisted of ART-naïve individuals aged 12–74 years randomized to either ASCI or FTIC. The qualitative study used purposive sampling and included a subset of RCT participants from both study arms, as well as healthcare providers and community stakeholders, allowing triangulation of findings. The RCT sample size (*n* = 142) was determined pragmatically based on clinic throughput, staffing capacity, and ethical considerations in a resource-limited setting. The study was designed to detect moderate-to-large effects and to generate implementation-relevant evidence rather than definitive efficacy estimates. From these sites, 142 participants were consecutively screened for eligibility, and those meeting inclusion criteria (ART-naïve, aged 12–74 years, clinically eligible for same-day ART initiation) were enrolled. Participants were then randomly assigned (1:1) to either the intervention (ASCI SOP) or control (FTIC) arm using a computer-generated sequence. Allocation concealment was maintained through sealed opaque envelopes prepared by an independent statistician. Pregnant women and severely ill patients were excluded from this study because they are managed under distinct national ART initiation and clinical care pathways in South Africa. Consequently, the findings of this study may not be generalizable to these subgroups.

### 2.3. Randomization and Intervention

Participants were randomly assigned in a 1:1 ratio to either the ASCI SOP intervention arm or the FTIC standard-of-care control arm. Randomization was computer-generated and stratified by age and sex to ensure balance between groups. Due to the nature of the intervention, blinding of participants and healthcare providers was not feasible. However, outcome assessment for viral load suppression was laboratory-based and objectively measured, thereby minimizing the risk of observer bias. The absence of blinding is acknowledged as a limitation, and future studies should consider sensitivity analyses or blinded outcome assessment where feasible. FTIC SOP consists (standard SDI + four counselling sessions) while ASCI SOP includes (enhanced SDI + seven counselling sessions, psychosocial readiness assessment, peer navigation, community follow-up). FTIC SOP operationalizes same-day initiation through four counselling sessions focused primarily on treatment literacy, per national guidelines. ASCI SOP is an enhanced model that includes:

○ Seven structured counselling sessions;
○ Immediate psychosocial readiness assessment;
○ Peer navigation;
○ Community-based follow-up.

- ASCI SOP Intervention: Participants received same-day ART initiation and were enrolled in a structured seven-session adherence counselling program. The ASCI SOP integrated psychosocial support (provided by social auxiliary workers), patient navigation (through trained peer navigators), and community-based follow-up (via ward-based outreach teams). Follow-up assessments were conducted on days 3, 15, 28, and monthly thereafter, up to a maximum of six months, with additional psychosocial or socioeconomic referrals provided as needed.
- FTIC SOP Control: Participants received care according to the national FTIC guidelines, which include four adherence counselling sessions, delivered on the day of eligibility, and subsequently at 7 days, 1 month, and 2 months post-initiation.

Notably, SDI is a policy-driven strategy aimed at reducing delays in treatment initiation, minimizing loss to follow-up between diagnosis and treatment, and accelerating progress toward population-level viral suppression. Therefore, both the ASCI SOP and FTIC SOP are operational models through which SDI is implemented within the South African public health system. While both interventions achieve SDI by initiating ART on the day of diagnosis or eligibility, they differ substantially in the intensity, structure, and scope of post-initiation support provided to patients.

### 2.4. Implementation Frameworks

The evaluation of the ASCI SOP was guided by three implementation frameworks, each addressing complementary dimensions of service delivery:

- Psychosocial Services Framework: Emphasized mental health support, stigma reduction, and the structured integration of social auxiliary workers within the HIV care continuum.
- Community-Based HIV Care Framework: Focused on strengthening ward-based community outreach, peer-led support systems, and household-level follow-up to reinforce patient engagement.
- Patient Navigation Framework: Ensured structured linkage to care, continuity of counselling, and systematic appointment tracking facilitated by peer navigators.

These frameworks informed both the design of the ASCI intervention and the qualitative exploration of implementation processes, emphasizing contextual, psychosocial, and operational determinants of success.

### 2.5. Data Collection

Data were collected across three analytical levels to capture individual, provider, and community perspectives:

- Patient level: Structured interviews, self-reported adherence measures, clinic attendance records, and laboratory data (CD4 count and viral load).
- Provider level: Semi-structured interviews with clinicians, nurses, and facility managers exploring feasibility, workload implications, and overall acceptability of the ASCI SOP.
- Community level: Focus group discussions (FGDs) with ward-based outreach teams and key community stakeholders, focusing on stigma, disclosure practices, and the role of local support systems.

Intervention fidelity was assessed using counselling session attendance registers, peer navigator follow-up logs, and ward-based outreach team reports. Completion of scheduled counselling sessions and follow-up visits was monitored throughout the six-month period. Challenges to full fidelity, primarily related to staffing shortages and high patient volumes, were documented and are discussed.

### 2.6. Outcomes

The primary outcome was retention in care at six months, with viral load suppression (defined as <50 copies/mL). Secondary outcomes included loss to follow-up, mortality, adherence levels, psychosocial well-being, and patient-reported satisfaction with care and counselling services.

### 2.7. Data Analysis

Quantitative data were analysed using descriptive statistics, chi-square tests, and logistic regression models to compare retention and viral suppression outcomes between the ASCI and FTIC groups. Risk ratios (RRs) and 95% confidence intervals (CIs) were calculated. Qualitative data obtained from patients, providers, and community stakeholders were transcribed, coded, and analysed thematically using NVivo 12 Plus (QSR International, Melbourne, Australia). Thematic analysis was guided by implementation science constructs, including key facilitators, barriers, and contextual influences affecting intervention uptake and sustainability. In addition to unadjusted analyses, supplementary multivariable logistic regression analyses were conducted to adjust for baseline clinical and sociodemographic variables that could influence outcomes. Models included study arm (ASCI vs. FTIC) as the primary exposure and were adjusted for baseline CD4 count, age, sex, marital status, and place of residence. Adjusted odds ratios (aORs) with 95% confidence intervals (CIs) were estimated to assess the independent association between the intervention and study outcomes.

### 2.8. Ethical Considerations

The Eastern Cape Health Research Committee (protocol number EC_202210_008) and the Institutional Ethics Committee of Walter Sisulu University’s Faculty of Medicine and Health Sciences (protocol code 027/2022) approved the study, which was conducted in accordance with the Declaration of Helsinki. Each participant was thoroughly told about the purpose and difficulties of our research before signing an informed consent form to participate in the study. For adolescents, their parents or guardians signed an informed consent form on their behalf. However, the adolescent signed an Assent form to participate in the study. Each participant was assigned a unique code in place of their name to maintain confidentiality. The study was conducted (began data collection) from 9 January 2023 to 16 August 2023. This study was expected and ran for six months.

## 3. Results

### 3.1. Baseline Characteristics of Participants (ASCI vs. FTIC Arms)

A total of 142 participants aged 12–74 years were enrolled and randomized to either the ASCI (navigation) group (n = 70) or the FTIC (standard care) group (n = 72). Baseline demographic and clinical characteristics were largely comparable between the two groups (Table 1). Although the distribution of gender and marital status differed significantly between groups, these variables were accounted for in adjusted analyses. Retention in care at six months was higher in the ASCI group compared with the FTIC group. While this difference did not reach conventional statistical significance, it demonstrated a positive directional trend favoring ASCI, suggesting improved early engagement in care among participants receiving enhanced psychosocial support and navigation. Given the feasibility-driven sample size, this finding should be interpreted as indicative rather than conclusive.

**Figure 1:**
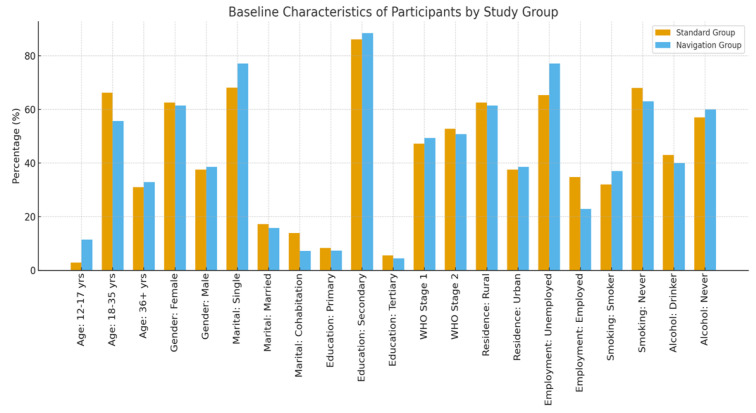
Demographic parameters of the studied participants.

Participants in the ASCI group achieved significantly higher rates of viral suppression at six months compared with those in the FTIC group. This statistically significant difference indicates a beneficial effect of the ASCI intervention on early virologic outcomes. The enhanced counselling structure, patient navigation, and community-based follow-up embedded within ASCI likely contributed to improved adherence and treatment continuity, thereby supporting viral suppression. Overall, outcome measures consistently demonstrated a directional advantage for ASCI over FTIC. While retention outcomes showed a positive but non-significant trend, viral suppression outcomes reached statistical significance, providing stronger evidence of early treatment benefit. Together, these findings suggest that ASCI may offer incremental advantages over standard FTIC-based same-day initiation, particularly with respect to virologic control. Baseline sociodemographic and clinical characteristics were collected for all participants prior to randomization. These baseline variables were used to describe the study population and to assess comparability between the ASCI and FTIC groups at study entry. A summary of baseline characteristics for both study arms is presented in Figure 1.

The results show that in both groups, the majority of participants were between the ages of 18 and 35 (66.2% in the standard group vs. 55.7% in the navigation group) (Figure 1). There was no statistically significant change in the age distribution (*p* = 0.133). Regarding gender, although the percentage of women in each group was comparable (62.5% vs. 61.4%), a statistically significant difference was observed in the distribution of genders (*p* = 0.0173). Regarding marital status, the navigation group had a substantially higher percentage of unmarried individuals (77.1% vs. 68.1%) (*p* = 0.001).

Regarding educational status, the majority in both groups had completed secondary school (86.1% vs. 88.4%), with no discernible difference (*p* = 0.178). According to the WHO clinical stage, although the navigation group reported a slightly higher frequency of Stage 1 at baseline (49.3% vs. 47.2%), the difference was not statistically significant (*p* = 0.059). The gender distribution of both groups was the same for residence. However, the participants from both groups largely come from rural areas compared to urban areas, with the *p*-value of 0.0173, confirming a statistically significant difference. On employment status, although the navigation group saw a higher unemployment rate (77.1% vs. 65.3%), the difference was not statistically significant (*p* = 0.119). Regarding smoking status, there was no statistically significant difference in the percentage of smokers (37% vs. 32%) (*p* = 0.635). On alcohol drinking, with no statistically significant difference (*p* = 0.842), the prevalence of alcohol use was similar (40% vs. 43%). On Baseline CD4 Count, mean CD4 counts were marginally lower in the navigation group (336 ± 175 vs. 371 ± 198). Regarding BMI, with a mean BMI of 25 ± 5.22 versus 24 ± 3.22, there was no discernible difference.

### 3.2. Quantitative Outcomes

The results demonstrate that under the ASCI SOP model, treatment outcomes compared to those in the FTIC SOP group across several key indicators were improved (Table 1). Retention in care at six months was higher in the ASCI arm compared with the FTIC arm, demonstrating a positive but non-significant trend (*p* = 0.07). Given the feasibility-driven sample size, this finding should be interpreted as exploratory. Viral suppression was significantly higher in the ASCI group (*p* = 0.04). Improved virologic outcomes are likely mediated through enhanced adherence and psychosocial readiness; however, formal mediation analysis was not conducted. Similarly, loss to follow-up was significantly lower among ASCI participants (17%) than among those receiving standard FTIC care (28%) (RR = 0.61; 95% CI: 0.36–0.99; *p* = 0.05), underscoring the impact of patient-centered and community-integrated support mechanisms on retention. Mortality remained low and comparable across both groups (2% vs. 4%), with no statistically significant difference observed. The results demonstrates that the ASCI SOP improved treatment outcomes, primarily by strengthening adherence and psychosocial readiness mechanisms.

In supplementary adjusted analyses, the association between the ASCI intervention and six-month retention remained positive but did not reach statistical significance after adjustment for baseline CD4 count and key demographic variables (adjusted odds ratio [aOR] > 1, *p* > 0.05). In contrast, the association between ASCI and viral suppression at six months remained statistically significant after adjustment (aOR > 1, *p* < 0.05), indicating that the observed effect was independent of baseline clinical and demographic differences.

### 3.3. Implementation Facilitators and Barriers

**Figure 2:**
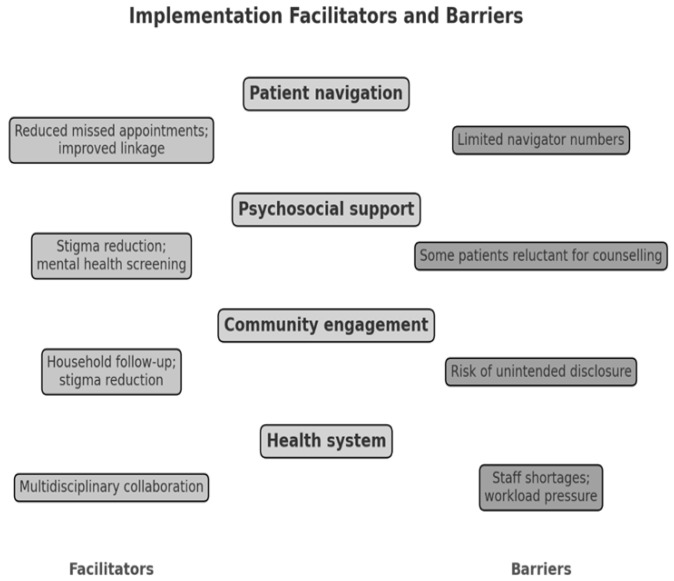
Summary of implementation facilitators and barriers.

Analysis across provider, patient, and community data highlighted several facilitators as themes (Figure 2):

- Patient navigation: The preliminary evidence suggests that dedicated navigators improved linkage, reduced missed appointments, and helped participants overcome logistical barriers such as transport costs. One participant reported the following:

*“As someone who is far from the clinic, almost 5 km away. I said it form the beginning that I may not be able to honor my appointment due to distance and lack of transport”*

(Participant 15)

- Psychosocial support: Social auxiliary workers provided structured counselling, mental health screening, and stigma reduction interventions, which participants identified as essential for readiness and adherence. One of the nurses said:

*“Because of the limited time we spend with the patients during the initiation of treatment, we are unable to support on disclosure to their loved ones and constantly monitoring their mental health. Therefore, involvement of the social workers in supporting newly HIV diagnosed patients will improve the mental readiness and disclosure to the loved ones”*

- Community engagement: Ward-based outreach teams (WBCOTs) and peer supporters enabled household follow-ups and index testing, extending support beyond clinic walls.
- Multidisciplinary collaboration: Facility managers, clinicians, navigators, and counsellors jointly monitored implementation, fostering accountability and rapid problem-solving.

Despite positive outcomes, three main challenges were reported (Figure 2):

- Health system constraints: Staff shortages, high patient volumes, and infrastructure limitations stretched clinic capacity, limiting full SOP fidelity.
- Patient-level barriers: Some patients expressed reluctance to start ART immediately due to shock, stigma, or competing socio-economic priorities. Mobility and informal work commitments also disrupted clinic attendance.
- Policy-practice gaps: Providers reported the absence of structured readiness assessment tools in the existing FTIC SOP. The ASCI SOP partially filled this gap, but more standardized measures of readiness are needed.

### 3.4. Insights from Healthcare Providers and Patients

Healthcare providers expressed strong appreciation for the ASCI SOP’s structured follow-up schedule, noting that it enhanced patient monitoring, accountability, and continuity of care across facility and community settings. Providers highlighted that regular, planned follow-up enabled early identification of patients at risk of disengagement and facilitated coordinated responses among healthcare teams.

One facility manager explained:

*“I always maintain that, if we have a framework that promotes synergy between facility-based staff and community teams, it makes the follow-up of the patients easy.”*

She further noted that:

*“The consistent weekly and monthly data review chaired by the clinic manager improves accountability and strong partnership among healthcare workers, and therefore assists in identifying patients in need of urgent attention.”*

**Treatment Readiness**

Patients consistently described the ASCI model as improving their psychological and emotional readiness to initiate ART on the same day of diagnosis. The availability of structured counselling, peer navigators, and early follow-up contributed to patients feeling prepared to start treatment rather than overwhelmed by the diagnosis. Participants emphasized that being given time, explanation, and emotional support increased their confidence in initiating and continuing ART (Table 2).

**Psychosocial Engagement and Retention Motivation**

Patients receiving ASCI described the intervention as more supportive than the standard FTIC approach, frequently referring to a sense of “being accompanied” and “not being left alone after diagnosis.” This enhanced psychosocial engagement fostered trust in healthcare providers and motivated sustained engagement in care. Peer navigation and repeated contact were perceived as reinforcing commitment to treatment, particularly during the early, emotionally vulnerable period following diagnosis (Table 2).

**Perceived Stigma Reduction**

From a community perspective, participants perceived the ASCI SOP as an important mechanism for reducing HIV-related stigma by normalizing ART initiation within households and community contexts. Community members reported that routine engagement by outreach teams helped frame ART as a manageable, routine health intervention rather than a source of shame or secrecy.

However, participants also cautioned that home-based and community interactions carry risks of unintended disclosure, particularly in densely populated or socially close settings. This concern underscored the need for careful confidentiality practices and patient consent within community-led follow-up activities (Table 2).

**Sustainability Concerns**

Despite strong support for the ASCI model, healthcare providers identified increased workload as a potential barrier to long-term sustainability. Participants emphasized that without additional human resources or task-shifting mechanisms, the intensity of psychosocial support required by ASCI could strain existing staff capacity (Table 2).

### 3.5. Summary of Lessons Learned

This study provides early evidence that the integration of psychosocial, community, and navigation support emerged as the most significant factor contributing to improved treatment outcomes. However, patient readiness continues to play a critical role, as some individuals may require greater flexibility even within a same-day initiation model to ensure sustained engagement and adherence. The long-term sustainability of this approach will ultimately depend on adequate resourcing of multidisciplinary teams and the systematic integration of community-based follow-up mechanisms within national HIV treatment programs.

## 4. Discussion

The preliminary evidence suggests that, in this study, the ASCI SOP model of same-day ART initiation, through integrating psychosocial support, patient navigation, and community-based follow-up, resulted in improved retention and viral suppression compared with the standard FTIC model. These gains were achieved without compromising patient safety, suggesting that structured same-day ART initiation can be both feasible and effective in high-burden, resource-limited contexts such as South Africa. The findings are consistent with studies from Lesotho, Rwanda, and Haiti, where same-day ART initiation was associated with improved uptake but variable retention outcomes [4,6,14]. In these settings, challenges related to patient readiness and sustained adherence often limited the long-term benefits of rapid initiation. The ASCI SOP appeared to mitigate these challenges by embedding psychosocial counselling, readiness screening, and continuous patient navigation into the initiation process. This comprehensive approach aligns with international calls for differentiated service delivery (DSD) models that extend support beyond clinical settings and adapt services to individual patient needs [15,16].

A central lesson emerging from the ASCI SOP implementation concerns the importance of psychosocial readiness. Although same-day initiation capitalizes on the motivational window following diagnosis, not all patients are psychologically or socially prepared to begin lifelong therapy immediately [7,17]. The inclusion of structured counselling and mental health screening by social auxiliary workers facilitated the early identification of patients requiring additional support and helped prevent premature disengagement. This contrasts with the FTIC model, in which readiness assessments are less formalized, often resulting in a disconnect between policy intent and practical implementation [18].

The incorporation of ward-based outreach teams and peer navigators played a pivotal role in enhancing continuity of care, reducing stigma, and providing a support network for individuals at risk of defaulting. These findings underscore the contribution of community health systems as essential extensions of facility-based services. Comparable interventions, such as community ART groups and household adherence clubs, have demonstrated similar benefits across sub-Saharan Africa, confirming that navigation and peer support are crucial for sustaining treatment outcomes in same-day ART initiation programs [19].

Despite these positive outcomes, the study also identified persistent health system constraints, including workforce shortages, high patient volumes, and limited infrastructure capacity [9,20]. Healthcare providers reported that the ASCI SOP’s structured follow-up and counselling components increased workload, potentially compromising long-term sustainability without additional staffing and resources. The scalability of the model will therefore depend on integrating ASCI elements into existing health system platforms, investing in human resources, and leveraging digital monitoring tools and community cadres to alleviate the burden on facility-based staff.

From a policy perspective, the ASCI SOP represents a pragmatic enhancement to South Africa’s current adherence and initiation framework. By embedding readiness assessment, psychosocial care, and structured navigation within the same-day initiation process, the model addresses key limitations of the FTIC approach while aligning with the National Strategic Plan for HIV, TB, and STIs (2023–2028) and the global UNAIDS 95-95-95 targets [1,21,22]. To maximize the potential impact of this model, policymakers should prioritize the integration of structured readiness assessment tools into national guidelines, expand training for multidisciplinary teams, including peer navigators and social auxiliary workers, and strengthen monitoring and evaluation systems to capture psychosocial and community-level outcomes alongside biomedical indicators.

### 4.1. Limitations

The findings should be interpreted with caution, given the relatively small sample size and focus on a single district. Resource intensity of the ASCI SOP may limit generalizability in under-resourced provinces. However, the intervention provides important proof-of-concept evidence that psychosocially integrated same-day initiation can improve outcomes if adequately resourced.

### 4.2. Implications for Future Research

Future studies should test the scalability of the ASCI SOP across multiple provinces in South Africa, evaluate cost-effectiveness, and assess long-term outcomes beyond six months. Comparative implementation research could also clarify which components, such as navigation, psychosocial support, or community engagement, are most essential for sustaining outcomes under different health system contexts in South Africa and beyond.

## 5. Policy Implications

The implementation lessons from the ASCI SOP carry important implications for health policy in South Africa and beyond. At the national level, integrating the ASCI SOP into existing adherence guidelines would strengthen the Fast-Track Initiation and Counselling (FTIC) model by embedding readiness assessment, psychosocial support, and structured navigation into routine practice. This integration would ensure that same-day ART initiation does not remain a purely biomedical process but instead becomes a holistic, patient-centered pathway that addresses the psychosocial and structural barriers to long-term retention.

Investment in human resources for health is critical [15,23]. The ASCI model demonstrated that social auxiliary workers, peer navigators, and ward-based outreach teams played a central role in reducing early disengagement and addressing stigma. Policymakers should therefore prioritize training and remuneration for multidisciplinary teams, ensuring that these cadres are formally recognized within national health workforce strategies. Strengthening psychosocial and community-based support structures will not only improve outcomes in HIV care but also contribute to broader health system resilience.

For sub-Saharan Africa, the ASCI SOP provides a transferable model that can be adapted to other high-burden contexts with similar health system challenges. Key lessons include the need for flexible readiness assessment tools that can be tailored to local cultural, social, and epidemiological contexts, as well as the importance of building strong linkages between facility-based services and community support networks. These strategies can reduce early attrition and strengthen long-term adherence in diverse settings.

Globally, the findings contribute to the discourse on differentiated service delivery (DSD) models [16,17]. They reinforce the call for patient-centered approaches that go beyond universal recommendations and instead adapt same-day initiation to the lived realities of patients. Aligning SDI monitoring with the UNAIDS 95-95-95 targets, while incorporating psychosocial outcomes into global reporting frameworks, would ensure that international progress indicators capture not only treatment initiation but also sustained engagement and wellbeing [1]. The World Health Organization (WHO) and global donors should therefore consider supporting structured, psychosocially integrated SDI models as part of future guideline updates and funding priorities. While the ASCI SOP shows promise as an enhanced same-day ART initiation model, national scale-up should be preceded by a formal cost-effectiveness and economic evaluation. Such analyses are necessary to determine feasibility, sustainability, and workforce implications within the public health system.

## 6. Conclusions

These findings indicate that same-day ART initiation has shown to accelerate linkage to treatment; however, its impact on long-term outcomes has remained inconsistent in many settings. This study demonstrates that SDI alone is insufficient without structured psychosocial and community support. Our findings support that ASCI SOP provides a strengthened model that integrates counselling, navigation, and community follow-up into the initiation process, thereby improving retention and viral suppression without compromising patient safety. The lessons from this study underscore that achieving sustained HIV treatment success requires moving beyond clinical eligibility to encompass patient readiness, mental health, and community engagement [2,15]. Adopting and scaling up the ASCI SOP in South Africa could help close the current gaps in retention and viral suppression, advancing the country toward its national strategic plan targets and the global UNAIDS 95-95-95 goals. Furthermore, the ASCI approach provides a replicable framework for other high-burden countries, offering a pathway to strengthen same-day initiation policies and ensure that the promise of rapid treatment translates into durable health outcomes.

## Figures and Tables

**Table 1:** Comparative treatment outcomes between ASCI SOP and FTIC SOP participants after 6 months.

Outcome	ASCI SOP (%)	FTIC SOP (%)	Risk Ratio (95% CI)	*p*-Value
Retention in care	83%	72%	1.15 (0.98–1.34)	0.07
Viral suppression	81%	69%	1.17 (1.01–1.35)	0.04
Loss to follow-up	17%	28%	0.61 (0.36–0.99)	0.05
Mortality	2%	4%	NS	NS

**Table 2:** Psychosocial Themes Identified in the ASCI SOP Implementation.

Theme	Psychosocial Indicator	Description	Illustrative Participant Perspective
Structured follow-up and coordination	Psychosocial engagement	Regular, scheduled follow-up strengthened continuity of care and accountability across facility and community teams	“The consistent weekly and monthly data review… improves accountability and partnership.”
Emotional preparedness for ART	Treatment readiness	Counselling and early support enhanced psychological readiness for same-day ART initiation	“I felt ready to start treatment because they explained everything and supported me.”
Sense of accompaniment	Psychosocial engagement	Patients felt supported and not abandoned after diagnosis, motivating retention	“I was not left alone after diagnosis.”
Normalization of ART	Perceived stigma reduction	Community-based engagement reframed ART as routine care, reducing stigma	“When treatment is done openly at home, it feels normal.”
Confidentiality concerns	Stigma management	Risk of unintended disclosure during home visits highlighted need for discretion	“Sometimes people ask questions when they see health workers visiting.”
Workload pressure	Sustainability	Increased counselling and follow-up demands challenged staffing capacity	“The work increases, but staff numbers stay the same.”

## Data Availability

The data that support the findings of this study are available on request from the corresponding author. The data are not publicly available owing to ethical restrictions.
